# A novel in silico Nuss procedure for pectus excavatum patients

**DOI:** 10.1007/s13246-023-01325-5

**Published:** 2023-09-11

**Authors:** Beop-Yong Lim, Dongman Ryu, Hoseok I, Chiseung Lee

**Affiliations:** 1 Department of Biomedical Engineering, Gradate School, and University Research Park, Pusan National University, Busan, 49241 Republic of Korea; 2 Medical Research Institute, Pusan National University, Busan, 49241 Republic of Korea; 3 Department of Thoracic and Cardiovascular Surgery, School of Medicine, Biomedical Research Institute, Pusan National University, Pusan National University Hospital, Busan, 49241 Republic of Korea; 4 Department of Biomedical Engineering, School of Medicine, Biomedical Research Institute, Pusan National University, Pusan National University Hospital, Busan, 49241 Republic of Korea

**Keywords:** Nuss procedure, Pectus excavatum, Finite element analysis, Computer simulation

## Abstract

The purpose of this study is to suggest a novel in silico Nuss procedure that can predict the results of chest wall deformity correction. Three-dimensional (3D) geometric and finite element model of the chest wall were built from the 15-year-old male adolescent patient’s computed tomography (CT) image with pectus excavatum of the mild deformity. A simulation of anterior translating the metal bar (T) and a simulation of maintaining equilibrium after 180-degree rotation (RE) were performed respectively. A RE simulation using the chest wall finite element model with intercostal muscles (REM) was also performed. Finally, the quantitative results of each in silico Nuss procedure were compared with those of postoperative patient. Furthermore, various mechanical indicators were compared between simulations. This confirmed that the REM simulation results were most similar to the actual patient’s results. Through two clinical indicators that can be compared with postoperative patient and mechanical indicators, the authors consider that the REM of silico Nuss procedure proposed in this study is best simulated the actual surgery.

## Introduction

Pectus excavatum (PEX) is a condition in which the person’s breastbone (or sternum) is sunken into the chest. In severe cases, PEX can look as if the center of the chest has been scooped out, leaving a deep dent [1]. While the sunken sternum is often noticeable shortly after birth, the severity of PE typically worsens during the adolescent growth spurt [2, 3]. Severe cases of PEX can eventually interfere with the function of the heart and lungs. But even mild cases of PEX can make children feel self-conscious about their appearance [4].

In order to correct PEX, the Nuss procedure is commonly carried out. The Nuss procedure inserts a curved metal bar through small incisions on each side of the chest. The bar is then flipped over to create an arch that presses upward on the sternum. In some cases, more than one bar is used [4]. The Nuss procedure is considered to be a representative minimally invasive surgery to correct PEX, however, if the PEX is severe or asymmetry, the procedure becomes very complicated. In particular, after puberty, the flexibility of the chest wall is decreased, requiring the insertion of two bars, making the procedure more difficult [5]. It also takes the patients longer to recover [6].

The precise correction of PEX through the Nuss procedure depends on the small insertion position and exact curvature of the metal bar to restore chest wall. Since the introduction of the Nuss procedure, various techniques to correct PEX have been proposed [7, 8]. In the short-bar technique, the bar may be guided manually through the chest wall, and no additional stabilizing sutures are necessary [9]. The use of additional bars should be considered to exert force on multiple sites of the thorax, allowing coverage of multiple rib levels [10]. Currently, there are two known surgical techniques: parallel bar insertion, and crossbar insertion using a double-bar insertion approach.

Various Nuss procedure techniques are subjectively applied to a in accordance with surgeons’ preferences. However, the fact that surgical methods are determined by preference is controversial. Surgical techniques using parallel bars or crossbars can cause significant constraints and pain in patients and breathing and motility may be affected by excessive insertion of metal bars. Therefore, several studies have been conducted to explore the optimal Nuss procedure, and some researchers have attempted to perform a in silico Nuss procedure, i.e., computational biomechanics-based virtual Nuss procedure [11–14].

In silico Nuss procedure studies, involving a three-dimensional (3D) geometry and finite element model (FEM) of the chest wall (consisting of the sternum, ribs, and costal cartilage), were constructed from computed tomography (CT) images of patients with PEX undergoing surgery. A simulation was performed in which a metal bar with varying curvatures was moved in the anterior direction under the sternum. The proposed optimal surgical indicators for individual patients included the curvature of the metal bar, the rib insertion locations, and the amount of anterior translation. Furthermore, simulations involving changes in the mechanical properties of the spine during the Nuss procedure, exploration of patients with both scoliosis and chest wall deformities, and the degree of pain reduction through stress during single- and double-bar-based Nuss procedures were investigated [15–17].

Previous studies have mainly focused on predicting clinical judgments of the Nuss procedure in a specific case through virtual simulation. On the other hand, studies aimed at solving the limitations and improvement of the Nuss procedure simulation are insufficient. Constantly moving a metal bar to the anterior of the chest and applying a simplified chest wall model composed of only the sternum, ribs, and costal cartilage were generally adopted as the process of Nuss procedure simulation [5, 18]. However, in actual surgery, a metal bar is placed concavely, inserted into the chest wall, and rotated to symmetrical with a convex metal bar. This action is restored the depressed sternum to treat PEX. At this time, after the rotational displacement of the metal bar, the chest wall and metal bar are generated a slight posterior movement to achieve physical equilibrium. In summary, in the actual Nuss procedure, the metal bar has a curved, frictional, and reactionary motion with rotational and equilibrium displacement, rather than a linear motion that moves anterior of the chest wall. Therefore, this study was expected that the applying rotational and equilibrium displacement similar to the actual surgery in the finite element analysis (FEA) had a small error.

**Fig. 1:**
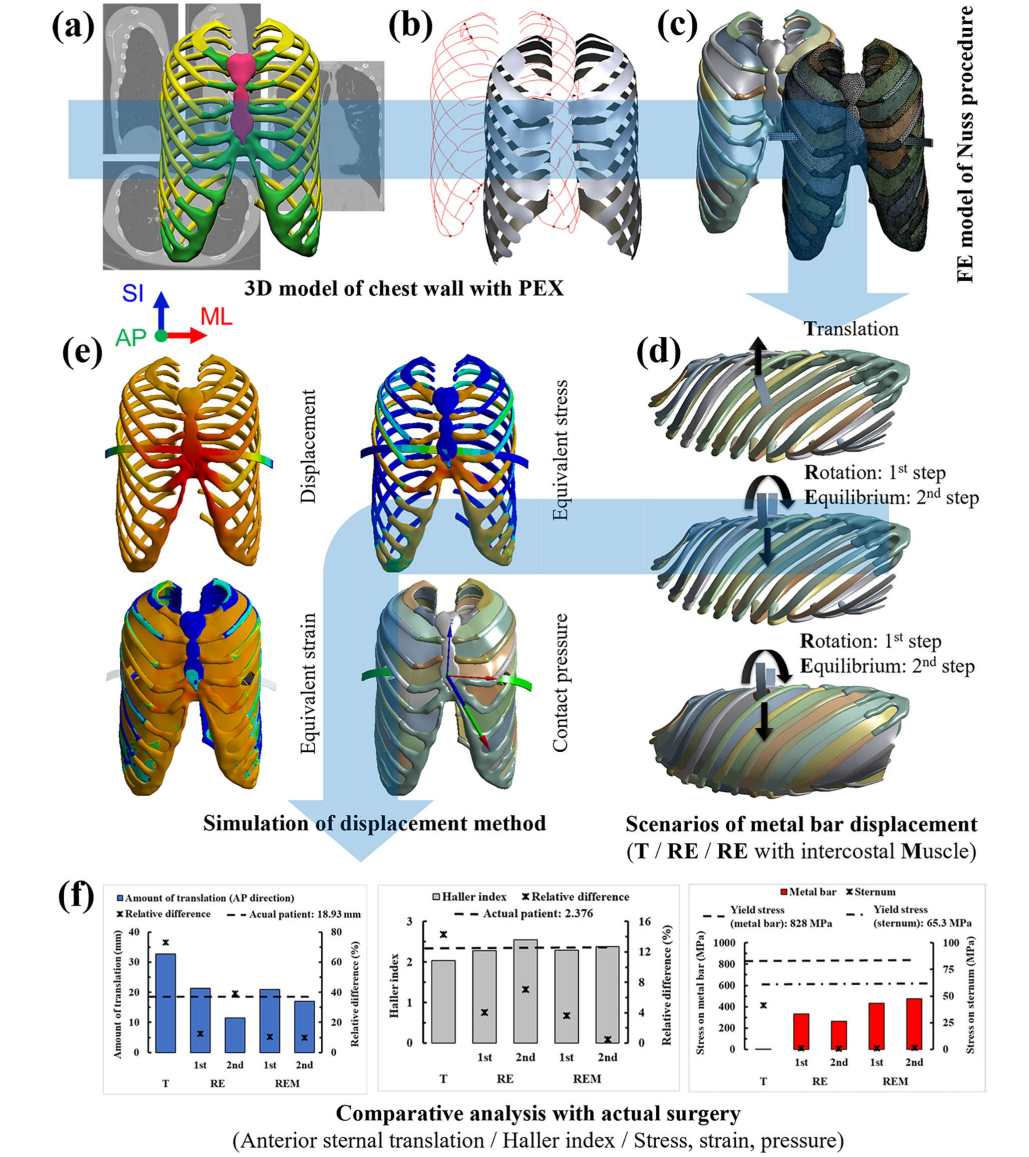
Overview of the Nuss procedure simulation according to the metal bar displacement method: **(a)** Extraction of a chest wall 3D model from the CT image; **(b)** Fabrication of the 3D model with ICM, defined as the plane connecting the centroids of adjacent ribs; **(c)** Construction of the chest wall and metal bar FEM; **(d)** Scenarios for the displacement of the metal bar; **(e)** Performance and result indicators of simulations. Analysis is performed for T, RE, and REM scenarios; **(f)** Comparative analysis with the results of the actual surgery

Therefore, the aim of this study is to increase the accuracy compared to the existing method after implementing the precise displacement of the metal bar and the delicate chest wall model in the in silico Nuss procedure. 3D geometric and finite element model of the chest wall were fabricated from the 15-year-old male adolescent patient’s CT image with pectus excavatum of the mild deformity. The material properties composed of ribs, costal cartilage, sternum, and intercostal muscles were loaded into the chest wall finite element model. After inserting the curved metal bar applied to the actual Nuss procedure into the 4th intercostal section, the in silico Nuss procedure was implemented to elevate the sternum by translating and rotating the metal bar. A simulation of anterior translating the metal bar (Translation (T) simulation), a simulation of maintaining equilibrium after 180-degree rotation (Rotation-Equilibrium (RE) simulation), and the RE simulation using the chest wall finite element model with intercostal muscles (Rotation-Equilibrium-intercostal Muscle (REM) simulation) were performed respectively. On the other hand, left-right rotation displacement may also be occurred after the rotation of the metal bar, but it is small compared to the equilibrium displacement. Therefore, it is assumed that only the main equilibrium displacement is occurred in this study. Finally, the quantitative results of each in silico Nuss procedure (i.e., Amount of anterior sternal translation and HI) were compared with those of postoperative patient. Furthermore, various mechanical indicators (i.e., equivalent stress and strain acting on the sternum and metal bar and contact pressure between the sternum and metal bar) were compared between simulations (Fig. 1). The accuracy and similarity of the in silico Nuss procedure and actual surgery can be quantitatively elucidated through this process. On the other hand, in silico surgery simulation through FEA is extensively studied not only on the chest wall, but also on the spine, joints, blood vessels, and lungs, thus securing considerable reliability [19–23].

## Methods

The FEA of the Nuss procedure proceeds in the following order: selecting various displacement controls of the metal bar, fabricating 3D models of the chest wall and metal bar, applying material properties to the detailed tissues of the chest wall and metal bar, and setting boundary conditions. To describe the movement of the actual chest wall and metal bar as closely as possible, emphasis was placed on the conditions for the anterior translational, rotational, and equilibrium displacement of the metal bar.

### Finite element model

To fabricate a 3D model of the chest wall, a medical image of the patient must be obtained. In patients with PEX, a typical type of symmetry was selected to reduce the error according to the unusual asymmetric type. A 15-year-old male patient visited the Pusan National University Hospital and underwent the Nuss procedure. The reason for selecting this patient was that most of them were children and adolescents in terms of the frequency of PEX by age. In addition, the frequency of PEX by type is because many symmetrical types are occurred. The tissues of the chest wall were separated and extracted from the preoperative CT images. Preoperative CT images taken at 2-mm intervals were selected for the convenience of 3D model fabrication. Among the tissues that comprise the chest wall, the sternum, ribs, costal cartilage, and intercostal muscle (ICM) have a mechanical effect on displacement control. In the CT images, tissues other than the ICM that could not be discerned were selected and extracted. The CT image was imported into Mimics 23.0 (Materialise, Leuven, Belgium), and each tissue was separated, masked, and three-dimensionalized. Early 3D models had rough surfaces and irregular holes; in this study, the surface was smoothed and filled because a rough surface lowers the convergence and accuracy of FEA.

The reason for considering the ICM was to simulate the interaction between the ribs more accurately. However, as the ICM could not be extracted from the CT images, it was assumed that the ICM existed based on the central location of the cross-section of the upper and lower ribs and costal cartilage. In the CT images of several ribs and costal cartilage, lines connecting the center of each section were drawn. Two central curves that matched the curvature of the ribs, and a square-shaped curved surface on both sides were constructed. This curved surface was defined as the ICM by giving it a certain thickness. The thickness of the ICM is approximately 1.97–4.85 mm according to the position difference of the anterior-posterior (AP) and superior-inferior (SI) [24]. Clinically, in this study, it was determined that the difference in muscle movement was small, and the average value was set equally to 3.39 mm.

A 3D model similar to the actual metal bar was produced for comparison with the results of the Nuss procedure. The 3D model of the metal bar was designed based on the height, width, thickness, and angle of the metal bar applied in the postoperative medical image (12.7 × 279.4 × 2.8 mm) [25]. The 3D model was designed using Inventor 2021 (Autodesk, Mill Valley, USA), a software suitable for the 3D design of mechanical models. The designed metal bar was inserted into the chest wall model containing the ICM. The insertion point of the metal bar model was designated as the height at which the chest depression started and at the 4th intercostal section, which is the insertion position of the metal bar applied to the actual patient. The insertion shape of the metal bar will differ depending on the displacement control method of the metal bar. In the anterior translation, the concave metal bar was inserted into the chest wall because the concave metal bar was moved linear motion without rotation. The center of the metal bar was placed in contact with the sternum in the thoracic cage. In the rotation, the concave metal bar was rotated within the chest wall and displaced in a convex shape, and the metal bar was placed in the chest wall in a concave shape at the position of the metal bar insertion described above. As the ICM of the chest wall was inserted to control rib movement, we decided to exclude the relationship between the ICM and the metal bar. Accordingly, the ICM at the point where the metal bar was inserted into the chest wall was removed by the radius of contact when the metal bar rotated.

The constructed 3D model of the chest wall and metal bar must be converted to FEM for analysis. The ICM was a surface type, and the remainder of the constitution was formed as a body type in the 3D model. Accordingly, the ICM was converted into an FEM consisting of two-dimensional shell elements, and the remainder was converted into a 3D tetrahedral or hexahedral element. Because the metal bar is a mechanical model based on exact dimensions, a hexahedral element was applied. As the sternum, ribs, and costal cartilage had a complex and irregular shape, a tetrahedral element was applied. To understand the impact of ICM, the results were derived by the model with and without ICM, respectively. The numbers of elements and nodes in the model without ICM were 162,362 and 307,781, respectively, and those in the model with ICM were 176,730 and 371,185, respectively (Table 1). In addition, the mesh sensitivity for this chest wall model has been established previously and is considered an appropriate form of FEM [25].

**Table 1:** Element type and number of elements and nodes for each tissue in the FEM of the chest wall

Tissues	Element type	Number of elements	Number of nodes
Sternum	Tetrahedron	4,907	8,333
Ribs	Tetrahedron	48,137	95,595
Costal cartilage	Tetrahedron	94,594	283,097
Intercostal muscle (ICM)	Square	28,760	92,398
Metal bar (Titanium)	Hexahedron	332	2,762
Total		176,730	371,185

### Material properties

Material properties should be accurately applied to understand the exact behavior of the constructed FEM. The model in this study consists of a chest wall and a metal bar, and the chest wall is made up of several tissues, such as the sternum, ribs, costal cartilage, and ICM. Additionally, the internal components of the sternum and ribs are divided into cortical and cancellous bones, with somewhat different material properties. The chest wall is deformed due to the Nuss procedure but is not fractured or excessively damaged. In addition, the metal bar was not deformed owing to its high elastic modulus. Accordingly, only the linear section was considered for determining the material properties of each tissue. The elastic modulus and Poisson’s ratio of each tissue were applied as described previously (Table 2) [26–29]. It was also assumed that all the tissues were homogeneous and isotropic.

**Table 2:** Elastic material properties of each tissue in the FEM

Materials		Young’s modulus (MPa)	Poisson’s ratio
Sternum	(Cortical bone)	11,500	0.3
	(Cancellous bone)	40	0.45
Ribs	(Cortical bone)	5,000	0.3
	(Cancellous bone)	40	0.45
Costal cartilage		37.5	0.3
Intercostal muscle (ICM)		10.3	0.3
Metal bar (Titanium)		200,000	0.29

As the sternum and ribs are divided into cortical and cancellous bones, this boundary should be clearly applied to the FEM. Because the cortical bone had a constant thickness surrounding the cancellous bone, it was formed on the sternum and ribs. The thickness of the sternal cortical bone was measured on the CT image of the patient, but the boundary of the costal cortical bone was unclear. Accordingly, the ribs were referred to in a previous study. The average thickness of the sternal cortical bone was 2.1 mm, and there was a slight difference in the thickness of costal cortical bone depending on the location. Therefore, 0.685 mm for manubrial, 0.7 mm for sternal, 0.725 mm for floating, and 0.685 mm for false were applied according to the location of ribs [30].

### Boundary conditions

In the Nuss procedure, the main boundary conditions affecting the chest wall are set by mechanical judgment by referring to the actual deformity of the chest wall. When the sternum is anteriorly translated by a metal bar, the manubrium of the sternum shows a relatively small amount of movement. The manubrium is restricted by the sternoclavicular joint where the manubrium and clavicle are in contact. Therefore, to derive the correct amount of anterior translation, the mechanical behavior of the sternoclavicular joint was analyzed, and controlled conditions were applied to the manubrium in contact with the clavicle [25]. When the metal bar is displaced, the spine and the most posterior part of the chest wall should be fixed to allow the sternum to translate. Accordingly, a fixed condition without movement was set at the costovertebral joint, where the vertebra and ribs were in contact. Separation between the tissues does not occur when the tissues of the chest wall are deformed. Therefore, the contact conditions between all the tissues were set as the bonded conditions. The metal bar was in contact with the sternum, causing displacement. In the anterior translation, the sternum and metal bar were in contact with each other and anteriorly translated simultaneously. Accordingly, the relationship between the two bodies was set as the bonded condition. In contrast, rotation is a movement in which the anterior translates while sweeping the sternum. At this time, a friction condition occurs between the two bodies and the friction coefficient should be determined. Several soft tissues exist in the anterior mediastinum and serve to significantly lower the coefficient of friction. In this study, because the coefficient of friction was very low, it was determined that this friction force had no significant effect on the mechanical behavior of the chest wall. Accordingly, a frictionless condition was set between the metal bar and sternum in the rotation.

By applying the established FEM, material properties, and boundary conditions, the analysis was performed according to the anterior translation, rotation-equilibrium, and rotation-equilibrium with ICM scenarios. The analysis was determined by structural analysis of large deformations by referring to the boundary conditions and degree of deformation of the chest wall. In this study, because the two displacements were sequentially performed in the equilibrium scenario, a multistep analysis was applied. The rotational displacement was described as 1st step, the equilibrium displacement as the 2nd step, and the confirmation of convergence of the equilibrium displacement as the 3rd step in scenario selection. These steps were performed sequentially, and the computational time was set to 1s per step for a total of 3s. ANSYS 2019 R1 (Ansys Inc., Canonsburg, USA) was used for the FEA, and the computer power was set to be suitable for various analysis conditions. The computer’s processor is an Intel(R) Xeon(R) CPU 2.40 GHz applied with 28 cores, and the RAM capacity is 64 GB.

### Physical quantities for validation of the virtual Nuss procedure

As a result of the FEA, the accuracy of each scenario was analyzed by comparing it with the results of patients who underwent the Nuss procedure. For comparison, each result was derived by selecting the anterior sternal translation and Haller index (HI) after the Nuss procedure. In addition, the equivalent stresses of the sternum and metal bars, which are the subjects of the behavior of the chest wall, were derived for the mechanical comparison analysis of each scenario. Finally, for quantitative comparison of each derived value, the relative difference between them was numerically expressed. The computational times for each scenario were as follows: Anterior translation: 0.05 h, Rotation-equilibrium: 10.90 h, and Rotation-equilibrium with ICM: 5.08 h.

After the Nuss procedure, the sternum was anteriorly translated, and the chest wall was restored close to the normal state. At this time, given that the sternum should be translated in an appropriate amount, the amount of anterior translation at the point where the metal bar and sternum are in contact is important. Therefore, the result of the actual Nuss procedure and the result of the scenarios in this study were compared to determine the amount of anterior sternal translation of the sternum. The distance between the same points preoperatively and postoperatively was measured based on the position at which the metal bar and sternum were in contact. The accuracy of each scenario was quantified by numerically expressing the relative difference between the measured value for each scenario and the measured value after the Nuss procedure.

In the case of chest wall deformities, such as PEX or pectus carinatum, the HI was used to numerically determine normality. The left/right lengths and anteroposterior length of the inner chest wall were measured in the section with the most severe deformation of the chest wall. The value obtained by dividing the left/right length by the anteroposterior length is the HI. The closer the HI after the Nuss procedure to the HI of the normal chest wall, the more accurate the result of the surgery [31]. The lengths of the chest walls were measured from the cross-sectional image of the actual patient after the Nuss procedure and the results of each scenario. The HIs were calculated using these values, and the accuracy was quantified by numerically expressing the relative difference between the calculated value of the actual Nuss procedure and the scenarios.

## Results

The displacement control methods for the metal bar considered in this study were anterior translational, rotational, and equilibrium displacements. To indicate the relative advantages of these methods more clearly, the scenarios were classified by selecting several criteria. The first criterion was the difference between anterior translation and rotation. Although the Nuss procedure used anterior translation in FEA, rotation is applied in reality; therefore, this difference should first be verified. Second, a displacement to achieve physical equilibrium occurs after rotational displacement, which is called the equilibrium displacement; accordingly, the difference between the rotation and equilibrium was selected as the criterion. Third, the difference due to the presence or absence of the ICM in equilibrium is used to check how the ICM affects rotation and equilibrium. Finally, because equilibrium proceeds in the order of equilibrium displacement after rotational displacement, the time point at which only rotational displacement was performed before equilibrium displacement was analyzed. To further clarify this, we named the rotational displacement in equilibrium as the 1st step and the equilibrium displacement as the 2nd step. In summary, each scenario was selected as anterior translation (T) / rotation-equilibrium (RE) (1st, 2nd step) / rotation-equilibrium with the ICM model (REM) (1st, 2nd step). Finally, to check whether the equilibrium displacement converges, we selected the 3rd step, to which the same displacement condition as the 2nd step is applied. This procedure also prove that the equilibrium displacement does not occur continuously after the 2nd step.

**Fig. 2:**
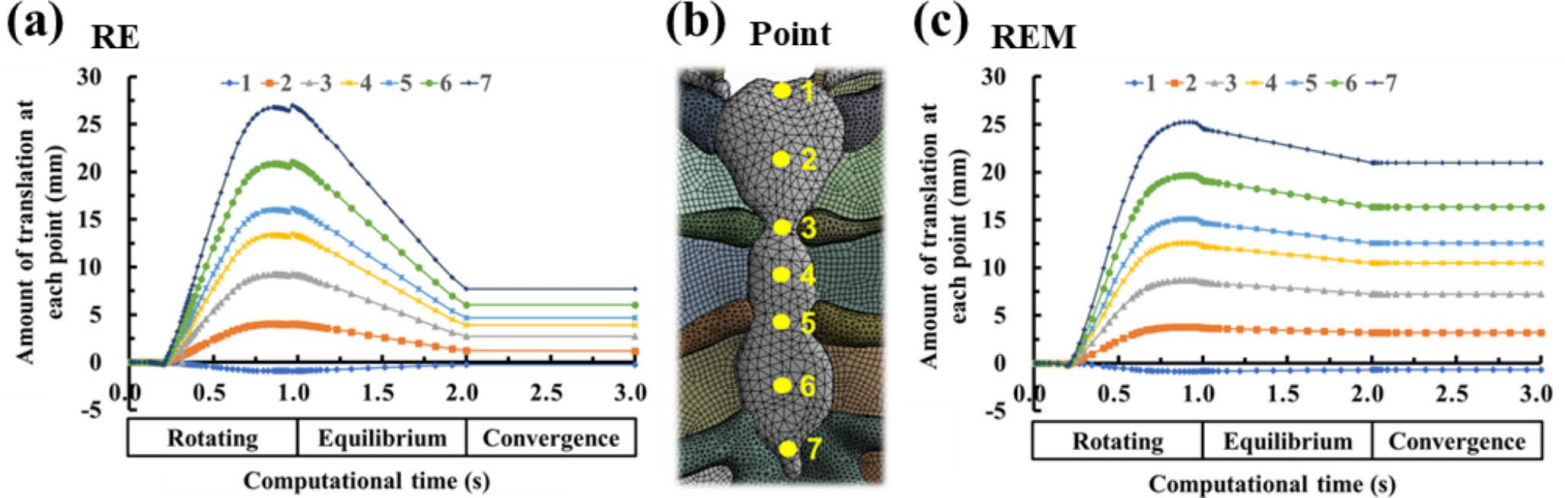
Amount of anterior sternal translation for each scenario according to the progress of computational time (1st, 2nd, and 3rd steps or rotating, equilibrium, and convergence, respectively): **(a)** Amount of anterior sternal translation over computational time in the RE scenario; **(b)** The point at which the anterior translation of the sternum is to be measured is marked; **(c)** Amount of anterior sternal translation over computational time in the REM scenario

Prior to the full-scale analysis of the results, there should be no further deformation of the chest wall after equilibrium displacement. To confirm this, the 3rd step for the convergence of equilibrium displacement was applied in the scenario selection and analysis setting. The amount of anterior sternal translation that could confirm changes in the chest wall from the FEA results by applying all steps was summarized by scenario. There was no significant change in the amount of anterior sternal translation in the 3rd step, and same physical quantity as in the result of 2nd step was derived; thus, there was no significant change in the chest wall after equilibrium displacement, and the equilibrium displacement converged (Fig. 2). Accordingly, the result of the 2nd step was proven to be the final chest wall change, and only the results of the 1st and 2nd steps were derived.

The characteristic observable part of the anterior sternal translation graph in the RE and REM represents the difference in change after the amount of anterior sternal translation reaches the maximum owing to the rotation of the metal bar. Both displacements started to decrease at approximately 0.8 s of computational time after the peak of anterior translation. However, a momentary upward point occurred during the descent, and a phenomenon of re-descent occurred in the RE. This was considered to have occurred because the cross-section of the metal bar had a right-angle shape when the metal bar was in contact with the posterior sternum. This does not occur during the REM, which is related to the force of the ICM to strongly restrict the chest wall. The REM was higher than the RE for the equivalent strain of the metal bar, and the metal bar of REM was relatively more spread. Therefore, it was concluded that RE characteristics were not expressed in REM.

### Comparison of anterior sternal translation between simulation results and clinical data

**Fig. 3:**
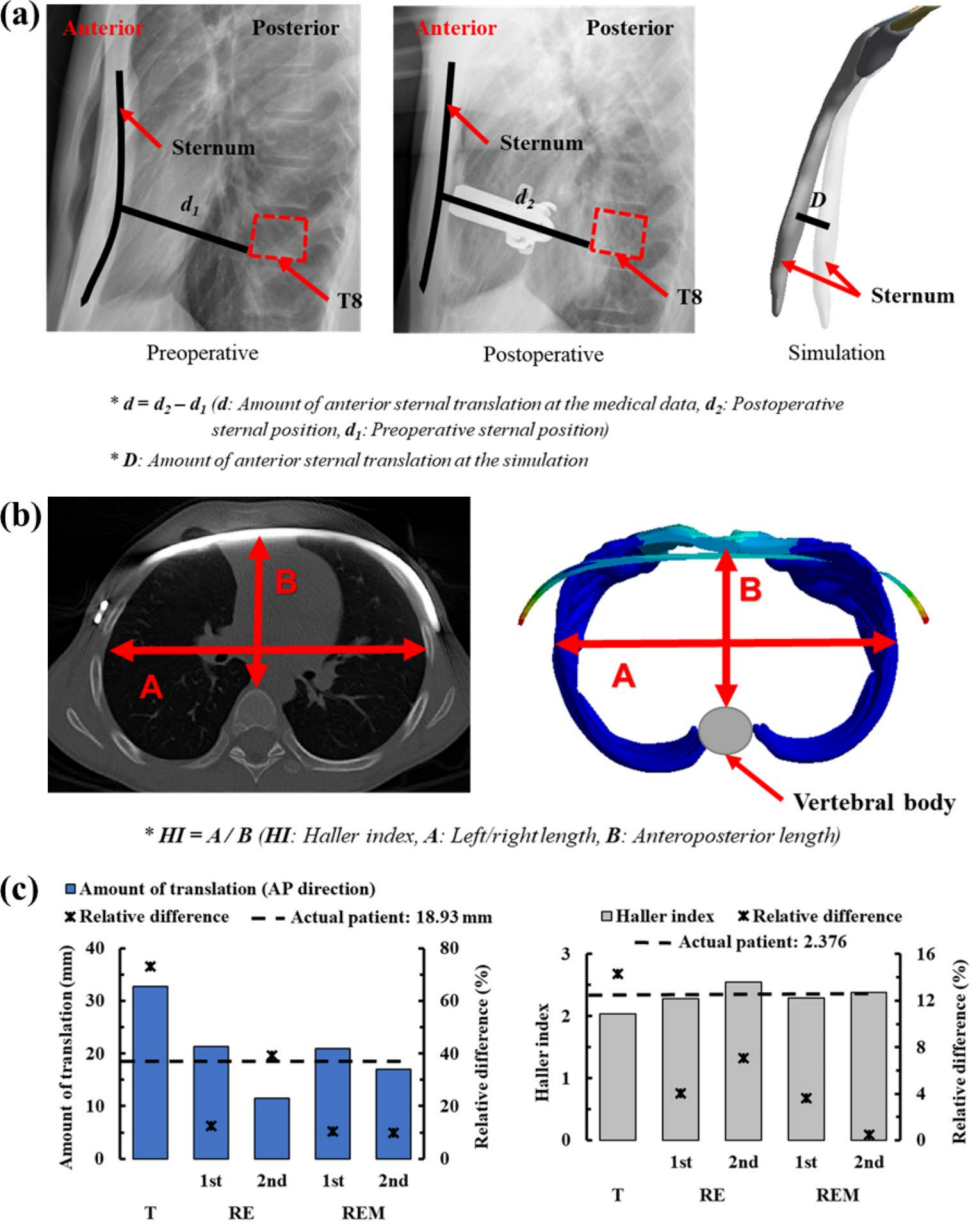
Comparison of the amount of anterior sternal translation and HI in the actual Nuss procedure and simulation scenarios: **(a)** Measurement of anterior sternal translation; **(b)** The HI represents the ratio of the left and right lengths in the chest cavity to the anteroposterior length in the cross-section of the chest wall; **(c)** Graphs showing the amount of anterior sternal translation and the HI of the actual Nuss procedure and each scenario

When comparing the relative difference value of the amount of anterior translational of each scenario with the actual Nuss procedure, the scenarios in which rotational displacement was applied were more accurate than T; this resulted in the relative difference of 73.06% compared to the actual value, but scenarios in which rotational displacement was applied resulted in relative differences of 9.83 to 39.25%. The derived values for the 1st step of RE and the 1st step of REM were not significantly different from the actual value. However, the difference in the derived values was large in the 2nd step of RE, depending on the presence or absence of ICM. Moreover, there was almost no effect of the ICM until the 1st step, but excessive posterior translation in the 2nd step occurred in the absence of ICM. However, a small amount of posterior translation occurred in the case of ICM, and equilibrium was established. As a result, the amount of anterior sternal translation was very close to the actual translation (Fig. 3).

**Fig. 4:**
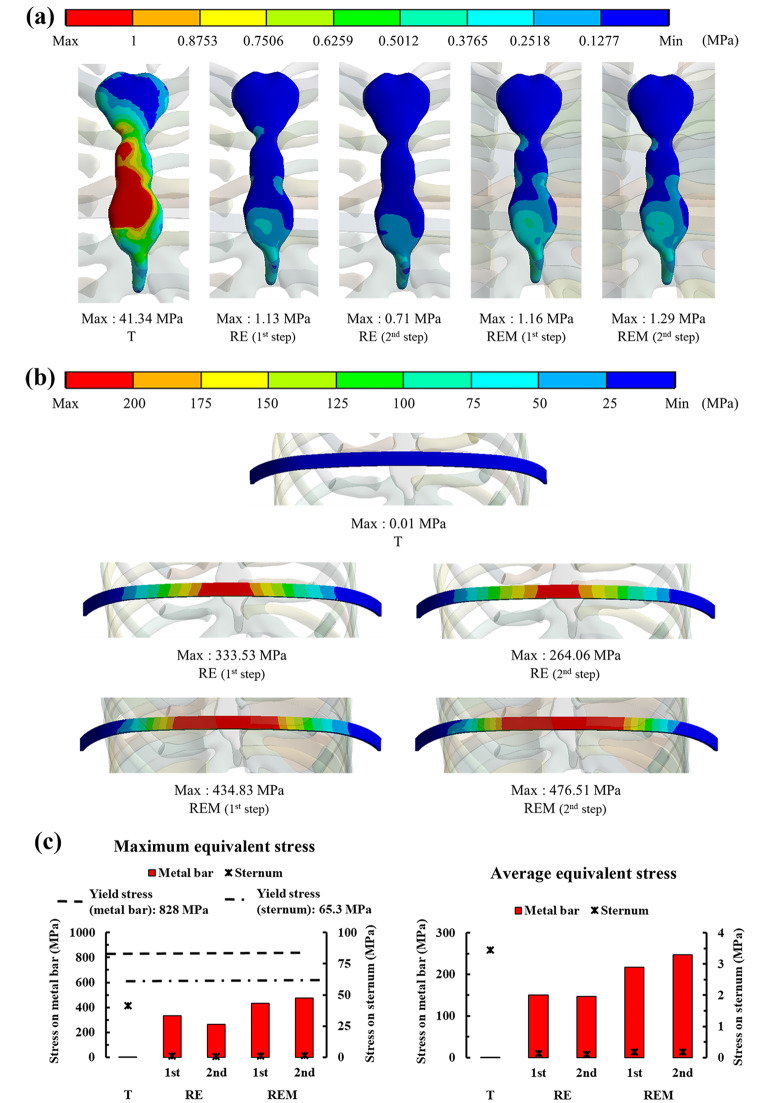
Maximum and average equivalent stress on the sternum and metal bar: **(a)** Equivalent stress distributions on the sternum by scenario; **(b)** Equivalent stress distributions on the metal bar by scenario; **(c)** Graph showing the maximum and average equivalent stresses on the sternum and metal bar for each scenario

**Fig. 5:**
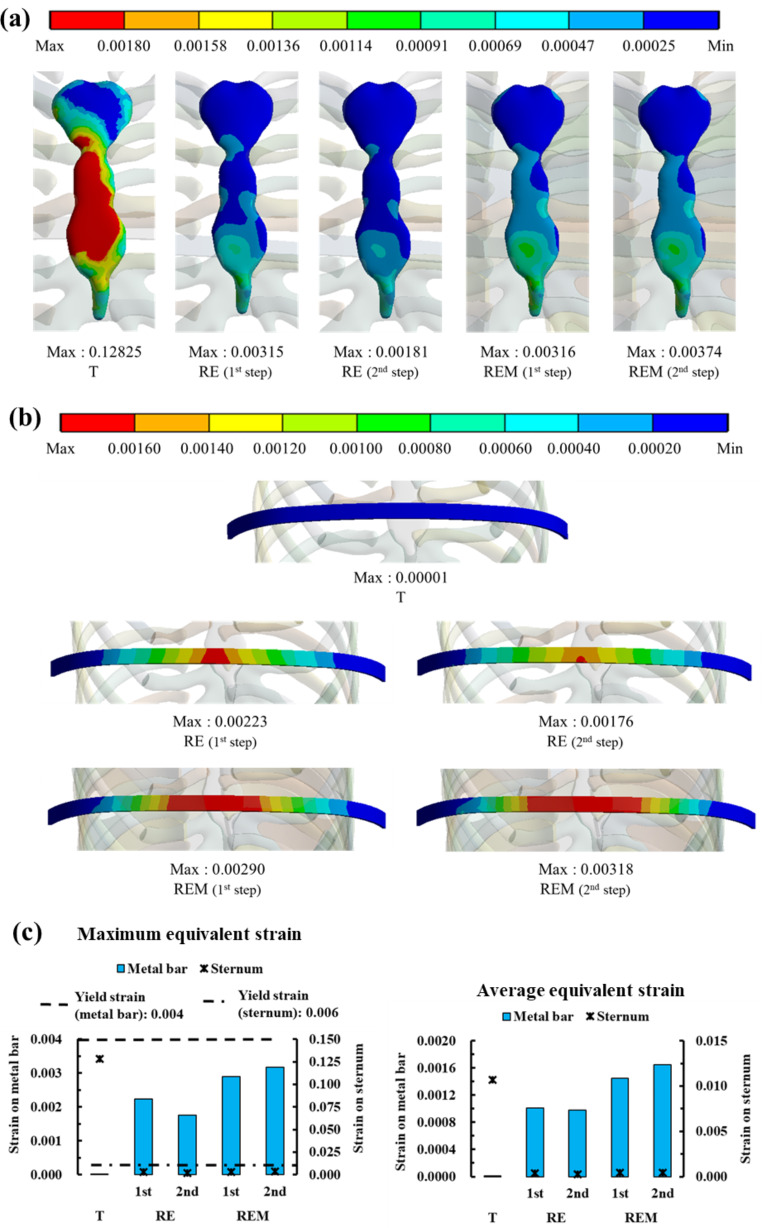
Maximum and average equivalent strain on the sternum and metal bar: **(a)** Equivalent strain distributions by scenario on the sternum; **(b)** Equivalent strain distributions on the metal bar by scenario; **(c)** Graph showing the maximum and average equivalent strains on the sternum and metal bar for each scenario

**Fig. 6:**
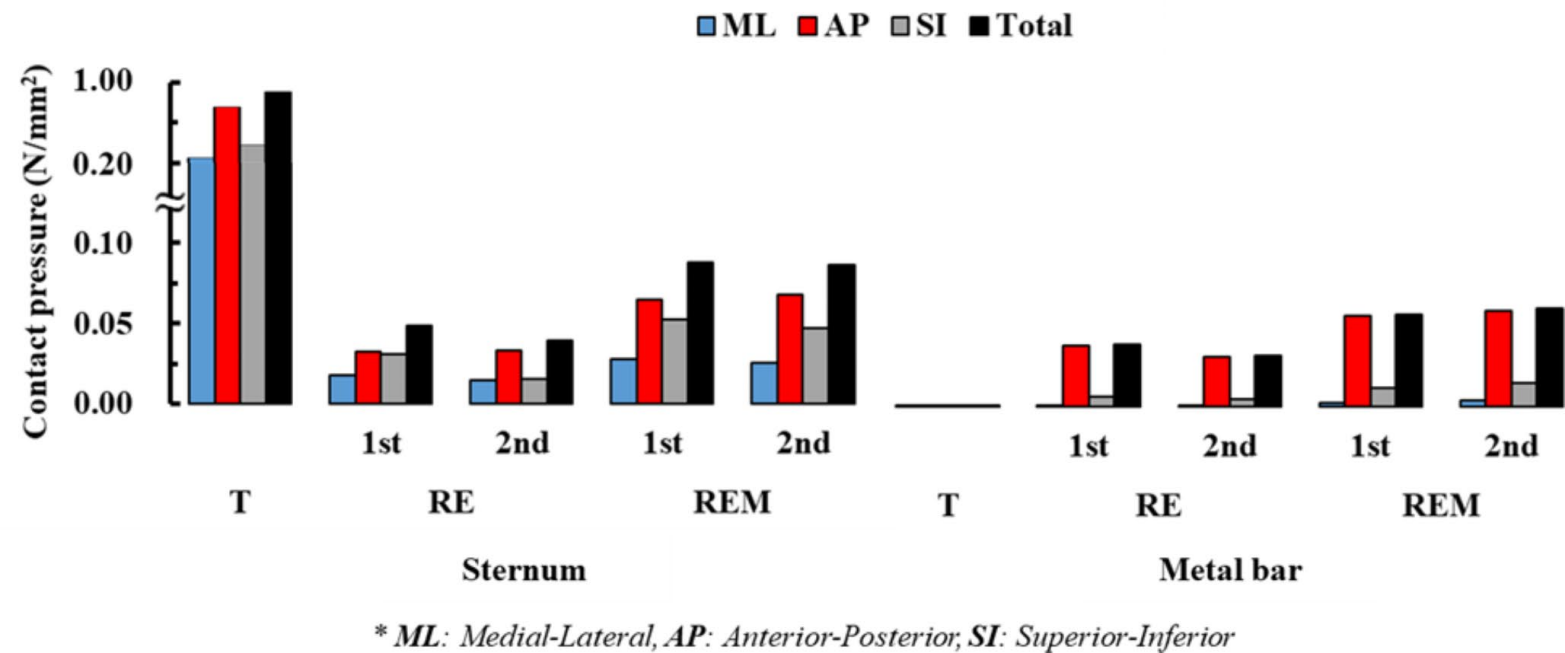
Contact pressure in the sternum and metal bar by scenario. The contact pressures for each scenario are displayed by dividing it into medial-lateral (ML), anterior-posterior (AP), superior-inferior (SI), and total summation according to the anatomical directional terms

### Comparison of haller index between the simulation results and clinical data

When comparing the relative difference in HI, the T was the most different, while the other scenarios were similar to the HI after the actual Nuss procedure. In particular, the 2nd step in REM showed the lowest relative difference and was close to the actual value. The HI was not particularly different in the scenarios excluding T, indicating that clinically similar surgical results were obtained. Therefore, the detailed displacement within the rotational displacement control of the metal bar does not have a significant effect on HI. However, there was a clear difference in the equilibrium, which was confirmed numerically (Fig. 3).

### Comparison of equivalent stress and strain between simulation results

The distribution of the equivalent stresses and strains on the sternum and metal bars was confirmed for each scenario. The physical relationship between the sternum and the metal bar was analyzed mechanically. Overall, the maximum equivalent stress of the sternum generated a stress of approximately 40 MPa in T and stresses of approximately 1 MPa in the remainder. The metal bar generated almost no stress, while the remainder generated stresses of approximately 260–470 MPa. The maximum equivalent strains also showed a similar tendency to that of the stress. However, in scenarios where rotational displacement was applied, the strains of the sternum and the metal bar were similar for each scenario. The average equivalent stress and strain also tended to be the same as their maximum values. However, in the average equivalent strain, the difference between the sternum and metal bar was approximately 2–3 times different in the scenarios where rotational displacement was applied (Figs. 4 and 5). To analyze the force generated at the point at which the sternum and metal bar contact, the contact pressure at that point was derived. The overall and individual anatomical directional term pressure was analyzed, and the pressures in the same direction from the sternum and the metal bar were compared. At T, the pressures were significantly different, and the scenarios in which rotational displacement was applied differed by approximately 1 to 2 times (Fig. 6). The yield stress and strain were investigated to determine whether plastic deformation of the sternum and metal bar occurred [32, 33]. The maximum equivalent stress and strain of the sternum and metal bars did not exceed the yield stress and strain in most scenarios. However, the equivalent strain of the T scenario on the sternum exceeded the yield strain; therefore, it was judged that the possibility of plastic deformation was high.

The patterns of the distributions and maximum values of the equivalent stress and strain were largely divided into T and rotational displacements. T caused higher stress and strain in the sternum, while the metal bar exhibited the opposite trend. The equivalent stress and strain of the sternum were almost the same within the rotational displacements, and the metal bar was changed according to the influence of the equilibrium and ICM. The equivalent stresses and strains of the metal bar increased overall compared to the RE in scenarios in which the ICM was applied. In the RE, the equivalent stress and strain of the metal bar decreased in the 2nd step compared to the 1st step. However, the stress and strain increased slightly during the 2nd step of REM.

## Discussion

Existing Nuss procedures in FEA studies have various mechanical problems that are different from those encountered in the actual Nuss procedure. Because the metal bar has rotational and equilibrium displacements in actual surgery, FEA assumes that the result would be more accurate if such displacements were applied. Several scenarios were selected in this study based on the characteristics of the various displacements. The scenarios were determined by dividing the T anterior translation, RE displacement in equilibrium after rotation, and REM applying the RE scenario in the ICM model. When the chest wall of PEX is restored to normal, the sternum, costal cartilage, and ribs determine the shape of the chest wall. In addition, the ICM has the greatest influence on the behavior of various ribs and costal cartilages. However, previous studies did not use a chest wall model with ICM. Accordingly, 3D models of the sternum, ribs, costal cartilage, ICM of the chest wall, and metal bars were constructed. For an accurate analysis of the Nuss procedure, material properties for each tissue, and boundary conditions to simulate the Nuss procedure were applied. In this study, we differentiated the material properties more precisely and applied additional boundary conditions for higher accuracy than has been possible in previous studies. To accurately apply the thickness of the cortical bone of the sternum and ribs, the thickness was measured directly from the CT image. In addition, because the thickness varied depending on the location of the ribs, different thicknesses were applied by dividing locations. Because the boundary condition of the previous study involved a fixed spine, only the fixational condition of the costovertebral joint was applied. However, as the sternoclavicular joint exists as a condition that limits the anterior translation of the sternum, this was additionally applied in this study.

After performing FEA for each scenario, the anterior sternal translation, HI, equivalent stress, strain, and contact pressure were derived from the FEA results. The displacement of the metal bar closest to the actual Nuss procedure was determined by comparing the derived physical quantity with the result obtained after the actual surgery and comparing each scenario. When measuring the amount of anterior sternal translation in each scenario, the relative difference in T was 99.47%, which was significantly different from the actual surgical result. However, the rotational displacements with RE and REM averaged 16.82%, which was closer to reality than T. In particular, the model with ICM was more accurate among the scenarios that applied equilibrium, and the relative difference in the 2nd step of the REM was most consistent (9.83%). Therefore, the REM is the most similar scenario based on the amount of anterior sternal translation. In addition, it was proven that ICM insertion is necessary when applying equilibrium displacement. The HI, which is related to the amount of anterior sternal translation, was most different from the actual surgery in the T group. The relative difference was 18.27% and the average of the rotation scenario group was 3.83%, which is close to reality. Again, the relative difference in the 2nd step of the REM was 0.46%, which was the most similar to the actual difference. Therefore, REM was the most similar to the actual results in terms of the amount of anterior sternal translation and HI, while T was judged to be significantly different from the actual surgery.

The T showed significantly lower equivalent stress and strain in the metal bar than in the rotation scenario group, which is the same as the result of the simple rigid movement of the metal bar of T. Therefore, this scenario differs from the chest wall behavior in the actual Nuss procedure from a mechanical point of view. The equivalent stress of T in the sternum was approximately 40 times higher than that in the rotation scenario group, while the equivalent stress T in the metal bar was approximately 400 times higher than that in the rotating scenario group. The difference in this stress multiple is due to the lower amount of sternal displacement in the rotation scenario group than that in the T scenario; this was due to the difference in material properties, such as the elastic modulus of the sternum and metal bar, and also provides a reasonable explanation for the difference in strain. REM had a higher equivalent stress and strain than RE without ICM because the ICM formed between the ribs controls the deformation of the chest wall and affects the metal bar. In addition, the 2nd steps of RE and REM showed different increases/decreases in the equivalent stress and strain compared to each step, which is also a result of dispersing the stress received by the rib to the ICM. Comparing the sternum and metal bar at the contact pressure in the AP direction, the 2nd steps of RE and REM values were most similar. This was reasonable from a mechanical point of view because equal pressure was applied to the two objects in contact. Based on this mechanical discussion, it was determined that REM follows the distribution of equivalent stress and strain close to reality.

Essential tissues were extracted from medical images of actual patients to construct a three-dimensional (3D) model of the chest wall. Tissues such as the sternum and ribs can be segmented automatically based on each Hounsfield unit. However, in the case of costal cartilage, automatic segmentation was not possible, and was instead performed from the researcher’s anatomical perspective. It is difficult and time-consuming to fabricate an accurate 3D model of the costal cartilage using this process. Since this limiting factor occurred similarly to other Nuss procedure studies, it is necessary to conduct additional studies or develop a method (such as by using CT equipment) that guarantees high-resolution images. Compared to T, the analysis time for the rotating scenario group was considerably longer owing to the nonlinear condition of contact between the metal bar and sternum in the rotation scenario group. If REM analyses were performed in a large number of patients, this difference would increase significantly. However, a simulation of the Nuss procedure is essential because it is difficult to apply the Nuss procedure to actual clinical practice in these studies. Therefore, it is necessary to save analysis time by using methods such as increasing computing power and 3D model simplification. PEX can be severe (such as asymmetric and eccentric PEX), and, depending on the type, various surgical methods can be employed, with alterations to the number, orientation, and position of the bars. However, as we analyzed the general type, further studies on other types are needed. In addition, the depression of the deformed chest wall after Nuss procedure should be appeared normal externally. However, the deformation of the skin which identifies the patient’s appearance was not considered in this study. Therefore, a study is required to confirm the appearance of the chest wall after the Nuss procedure simulation. As a limitation, the reasons for not applying nonlinear material to the material properties of chest wall tissue were given in the methods section. However, it is considered that more accurate results can be obtained if non-linear material is applied to costal cartilage. matters related to the connection between the metal bar and ribs and time-dependent analysis were not considered. Future studies should examine the safety of metal bars through creep analysis, as well as by considering the fastening method of the metal bar and ribs, and the type of suture.

## Conclusion

Several FEA studies have been conducted using the Nuss procedure, and most have derived stress on the chest wall by simply translating the metal bar [15–17]. However, if rotational and equilibrium displacements are applied rather than anterior translation of the metal bar, as in this study, it is expected that there will be a significant difference from the stress results of previous studies. In conclusion, because the exact application of the displacement on the metal bar can have a significant influence on the results, this study presents an accurate process for FEA studies of the Nuss procedure. Additionally, if this technique is referred to in clinical studies, it is expected to provide expandability by analyzing more diverse patients and detailed surgical methods.

The novel Nuss procedure simulation method presented herein describes the actual Nuss procedure more precisely than the existing ones, and the resulting chest wall shape is also more accurate than the actual one. This was conducted to mechanically evaluate the mechanism of the Nuss procedure simulation by quantitatively deriving the physical effect of the metal bar displacement method on the chest wall. This simulation method can be used clinically by presenting quantitative and clear judgment criteria for performing the Nuss procedure. It is also possible to provide the results to the clinician and patient in advance through the accurate prediction of the chest wall after the Nuss procedure. This utilization will impact the surgeon’s planning, and technical improvement in the surgical method can be expected.

Several scenarios were assumed according to the displacement of the metal bar in the Nuss procedure, including anterior translation, rotation, and equilibrium. FEA was performed based on these scenarios and compared with the actual surgical results. It was determined that the equilibrium displacement after rotation in the ICM model was most similar to the actual condition. If the limitations outlined in the Discussion section are improved and simulations applying various methods for additional metal bars are conducted, the possibility of application to actual clinical practice is expected to increase. This study suggests a new condition for the metal bar displacement in the FEA study of the Nuss procedure, which will improve the accuracy of computational biomechanical studies and predictability for clinical studies of the Nuss procedure.
